# Diagnostic Accuracy for Per-Patient Polyp Detection of Second-Generation Capsule Endoscopy Compared to Colonoscopy: A Meta-Analysis of Multicenter Studies

**DOI:** 10.7759/cureus.17560

**Published:** 2021-08-30

**Authors:** Hassam Ali, Rahul Pamarthy, Shiza Sarfraz, Eslam Ali

**Affiliations:** 1 Internal Medicine, East Carolina University, Vidant Medical Center, Greenville, USA; 2 Anesthesiology, Bahawal Victoria Hospital, Quaid-E-Azam Medical College, Bahawalpur, PAK; 3 Gastroenterology, East Carolina University, Vidant Medical Center, Greenville, USA

**Keywords:** cce, colon capsule endoscopy, colonoscopy, colon cancer prevention, colon cancer survillence

## Abstract

Colon capsule endoscopy (CCE) or capsule colonoscopy can be used as colorectal cancer (CRC) screening option. We intended to analyze the concerning literature that compared second-generation CCE to standard colonoscopy for multicenter studies only. A literature search was performed in PubMed, Embase, and Web of Science. Study characteristics related to our research including sensitivity and specificity for per-patient polyps detection (size: ≥ 10 mm and ≥ 6 mm). Meta-analysis was performed using an open meta-analyst. Our research included five studies, involving a total of 1518 patients, with a total of 1305 analyzed patients. The adequate bowel preparation rate ranged from 70% to 90%. The rates of complete CCE transit fluctuated from 80% to 100%. Our meta-analysis illustrated that mean (95% confidence interval) per-patient sensitivity, specificity, and diagnostic odds ratio were: 0.86 (0.82-0.91) (p < 0.001), 0.88 (0.72-0.96) (p < 0.001), and 50.7 (18.5-138.9) (p < 0.001), respectively, for polyps ≥ 6 mm; and 0.86 (0.8-0.91) (p < 0.001), 0.96 (0.92-0.98) (p < 0.001), and 173.5 (98.4-305.8) (p < 0.001), respectively, for polyps ≥ 10 mm. We concluded that CCE had high sensitivity and specificity for per-patient polyps vs. standard colonoscopy. Nevertheless, the comparatively higher rate of unfinished CCEs limits the utilization of CCE for CRC screening.

## Introduction and background

More than 1.9 million new colorectal cancer cases and 935,000 deaths occurred in 2020, representing about one in ten deaths secondary to cancer. Overall, colorectal cancer (CRC) ranked third in terms of incidence and second in terms of mortality per GLOBOCAN-Global Cancer Statistics 2020 [1]. CRC is a preventable and treatable disease, especially if recognized in the early stages. Primary prevention entails lifestyle and dietary modifications. Secondary prevention in asymptomatic individuals (screening) is recommended at ages 45 and older [2]. Colon capsule endoscopy/capsule colonoscopy (CCE) utilizes a small capsule to observe the mucosa of the colon and rectum. It is a minimally invasive screening technique with the principal advantage being a painless procedure without the risk of complications associated with standard colonoscopy (SC); they do however pose a risk of retention or intestinal obstruction. CCE was first introduced in 2001 [3]. Second-generation CCE is currently the latest development in capsule colonoscopies. Thus, this meta-analysis aimed to summarize the multicenter studies examining the diagnostic accuracy of second-generation CCE compared to colonoscopy in detecting colorectal polyps. Previously, only one metanalysis has been conducted including single-center trials with a high risk of bias [4]. Our aim was to utilize only multicenter trials to reduce the risk of bias when comparing CCE to SC.

## Review

Materials and methods

This meta-analysis was performed and reported per the PRISMA (preferred reporting items for systematic reviews and meta-analyses) guidelines [5].

Literature search

**Figure 1:**
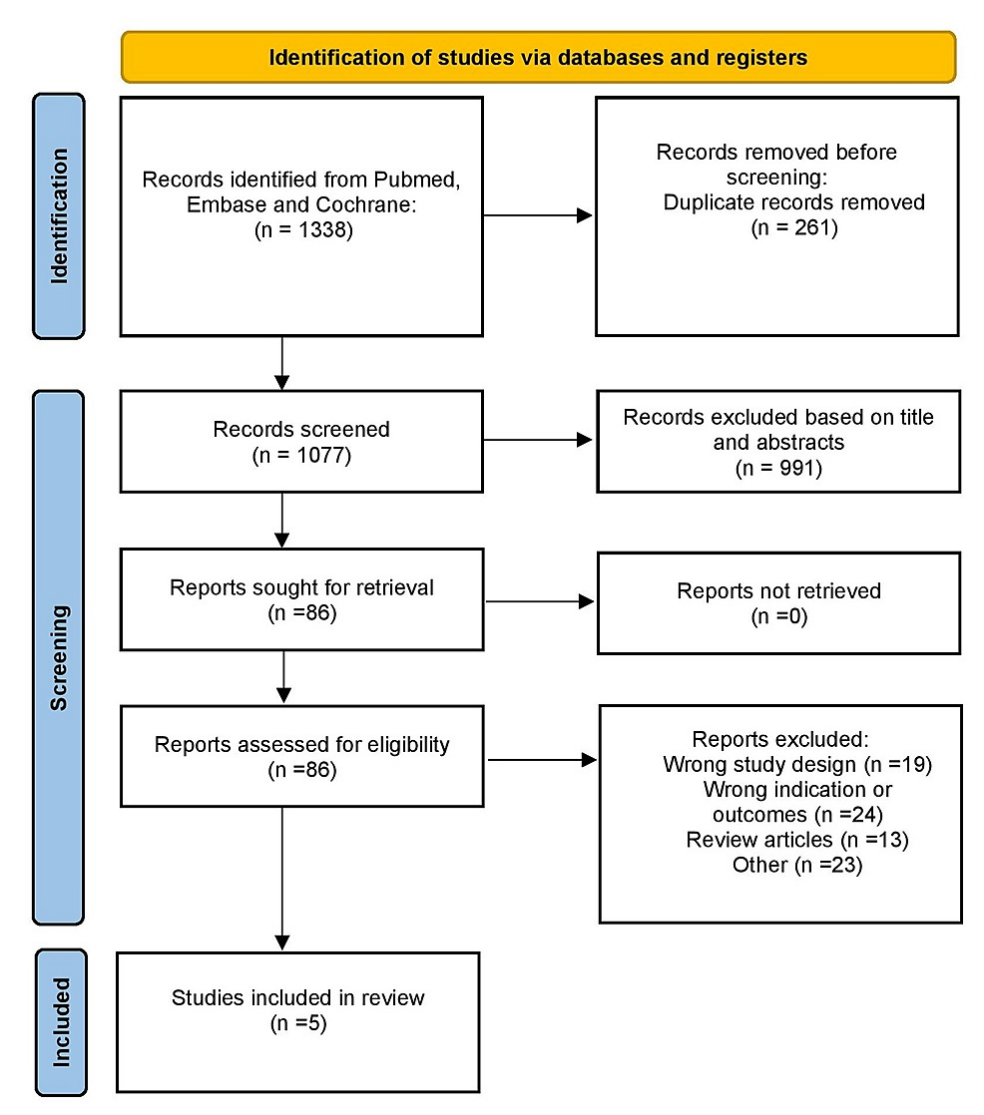
PRISMA flow chart PRISMA: preferred reporting items for systematic reviews and meta-analysis

A systematic literature search of PubMed, Embase, and Cochrane Library was performed on 30th March 2021 to distinguish research papers investigating the diagnostic test accuracy of capsule colonoscopy to standard colonoscopy. The PRISMA flowchart is given in Figure 1. Titles and abstracts were screened to identify studies comparing CCE and colonoscopy for patients undergoing both procedures. Full texts were read thoroughly by two reviewers. A third reviewer resolved disagreements. The data extracted from the studies included true positives, false positives, true negatives, false negatives, sensitivity, and specificity for the following outcomes: polyps (≥ 10 mm, ≥ 6 mm). Study characteristics also included data regarding the comparison of standard colonoscopy. The authors include the number of patients, age, colonoscopy indications, bowel preparation, and their quality and CCE transit percentages. 2x2 tables were utilized to summarize components of sensitivity and specificity and polyp sizes. We did not summarize any adverse effects. We used the Quality Assessment of Diagnostic Accuracy Studies tool to assess the risk of bias [6]. This has been summarized in Table 1 [7-11]. We found that two studies had a lower bias risk concerning patient selection [8,11]. All studies included in our meta-analysis except Parodi et al. classified SC as high risk of bias [8].

**Table 1:** Bias assessment for studies included in our meta-analysis CCE: Capsule colon endoscopy; SC: Standard colonoscopy

	Patient Selection		Index test (CCE)		Reference (SC)		Flow and timing
Study	Bias	Applicability	Bias	Applicability	Bias	Applicability	Bias
Eliakim 2009 [7]	High	Low	Low	Low	High	High	Low
Parodi 2018 [8]	Low	Low	Low	Low	High	High	Low
Rex 2015 [9]	High	Low	Low	Low	High	High	Low
Spada 2011 [10]	High	Low	Low	Low	High	High	Low
Voska 2019 [11]	Low	Low	Low	Low	High	High	Low

Statistics

Statistical analyses were performed in open meta-analyst 12.11.14. A random-effects model (REML) was deemed appropriate to produce unbiased estimates of variance parameters considering the variation in inclusion criteria for the five included studies. Summary points and 95 % confidence intervals (CIs) were calculated for per-patient sensitivity, specificity, and diagnostic odds ratio for patient outcomes. Forest plots were obtained to present the results graphically.

Results

We performed a meta-analysis for polyps ≥  10  mm, and polyps  ≥  6  mm. Five studies with a total of 1305 (1518) patients were included in this meta-analysis [4-8]. Study characteristics have been described in Table 1. Polyethylene glycol combined with sodium phosphate (PEG + NaP) were the most common bowel preparation agent. The average rate of adequate bowel preparation was 82% (Table 2). The average rate of complete CCE transit was 91.6% of all capsules ingested. 

**Table 2:** Characteristics of studies included in our analysis CRC: Colorectal Cancer; FIT: Fecal Immunological Test; PEG: Polyethylene Glycol; NaP: Sodium Phosphate

Study, year	Study type	Total Patients (Analyzed patients)	Mean age,years	Indications for colonoscopy	Bowel preparation	Adequate bowel preparation %	Complete CCE transit %
Eliakim 2009 [7]	Multicenter	104 (98)	50	CRC screening History of polyp/CRC	PEG + NaP	78	81
Parodi 2018 [8]	Multicenter	177 (177)	57	First-degree relatives to patients with CRC	PEG + NaP	81	100
Rex 2015 [9]	Multicenter	884 (695)	57	Screening	PEG + Suprep	80	92
Spada 2011 [10]	Multicenter	117(109)	60	CRC screening History of polyp/CRC	PEG + NaP	81	88
Voska 2019 [11]	Multicenter	236 (225)	59	Screening	PEG + NaP	90	89

**Figure 2:**
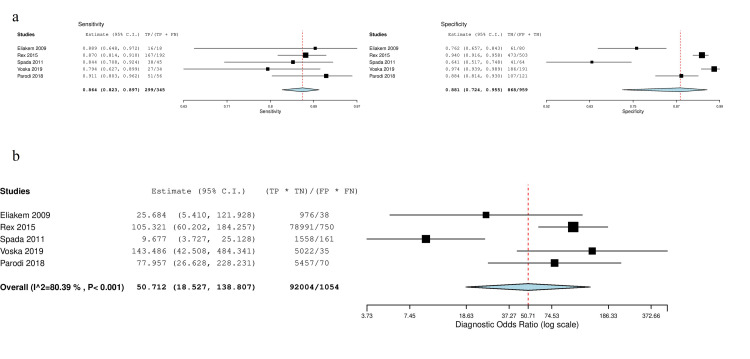
Forest plots for polyps ≥ 6 mm. a: Sensitivity and specificity, b: Diagnostic odds ratio TP: True Positives; FP: False Positives; FN: False Negatives; TN: True Negatives; CI: Confidence Intervals.

For polyps  ≥  6  mm, the mean (95 % CI) per-patient sensitivity, specificity, and diagnostic odds ratio were 0.86 (0.82-0.91) (p < 0.001), 0.88 (0.72-0.96) (p < 0.001), and 50.7 (18.5-138.9) (p < 0.001), respectively. For diagnostic odds ratio, τ2 was 1.016 and Cochran’s Q was significant (< 0.001 ). Forest plot for polyps ≥  6  mm sensitivity/ specificity is presented in Figure 2a. Forest plot for diagnostic odds ratio is shown in Figure 2b.

**Figure 3:**
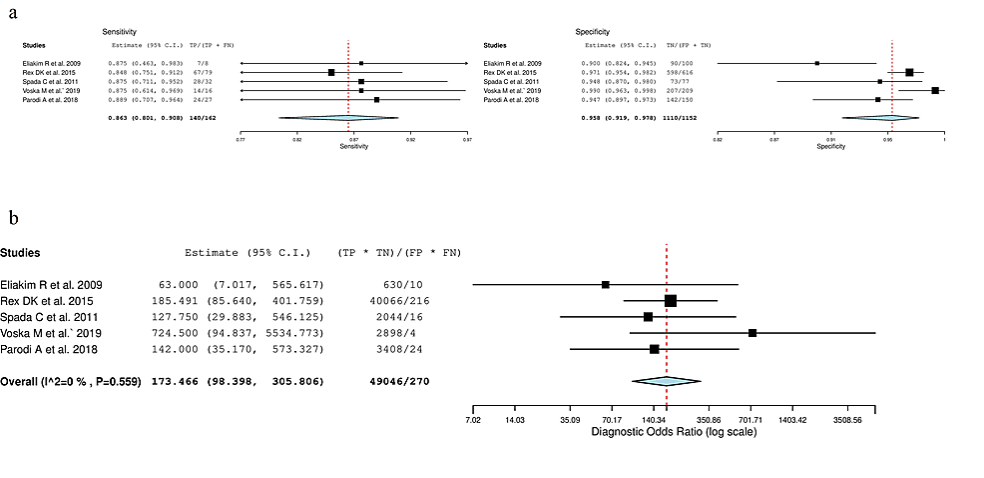
Forest plots of polyps ≥ 10 mm. a: Sensitivity and specificity, b: Diagnostic odds ratio TP: True Positives; FP: False Positives; FN: False Negatives; TN: True Negatives; CI: Confidence Intervals.

For polyps  ≥  10  mm, the mean (95 % CI) per-patient sensitivity, specificity, and diagnostic odds ratio were 0.86 (0.8-0.91) (p < 0.001), 0.96 (0.92-0.98) (p < 0.001), and 173.5 (98.4-305.8) (p < 0.001), respectively. For diagnostic odds ratio, τ2 was 0.000 and Cochran’s Q was nonsignificant (p =  0.559). Forest plot for polyps ≥  10  mm sensitivity/specificity is presented in Figure 3a. Forest plot for diagnostic odds ratio is shown in Figure 3b.

Discussion

Several randomized control trials exist for the investigation of second-generation CCE in detecting colorectal carcinoma versus standard colonoscopy. Capsule endoscopy can be a bridge between other forms of CRC screening and therapeutic colonoscopy. Limited data exist regarding the newer second-generation capsule endoscopy's utility in detecting CRC. Only one meta-analysis was done previously, which also included single-center trials, leading to low generalizability and a high risk of bias [4]. Our meta-analysis included only multicenter trials and revealed higher per-patient sensitivity and specificity for detecting polyps ≥ 6 mm, and ≥ 10 mm. In the reported trials, one of the main issues was incomplete transit through the colon due to the limited battery capacity of capsule endoscopes [7-11]. If capsule colon endoscopy can be used for CRC screening, it can lead to fewer diagnostic colonoscopies, reducing complications associated with SC. CCE can be employed as a filter test between fecal immunochemical test (FIT) and standard colonoscopy. The use of CCE depends on the number of screening colonoscopies that could be bypassed by this test. It would also benefit younger patients who need screening colonoscopies with probable negative results since now recommend screening at 45 years [2]. In the studies included in our meta-analysis, the bowel preparation regimens were adequate and comparable. The percentages of bowel preparations that were reported ranged from 70% to 90%. In these multicenter studies, the average completion rate of colon capsules was above 90%. For capsule endoscopy, proper bowel preparation must be ensured to aid the completion of capsule transit. Studies have reported improved capsule transit times depending on the adequacy of bowel preparation. One study reported that bowel preparation with castor oil could be a viable option in improving capsule ejection rate [12]. The potential implementation of CCE in CRC screenings in the United States also demands the capsule's cost and the data analysis. Standard colonoscopy is also linked to variable results in terms of polyp size [9]. CCE can visualize the colon without the requirements needed for colonoscopy, for example, sedation [7]. The data recorder and analytic software help smarter analysis, and the capsule can measure up to 35 images per second, giving it an advantage when passing through rapid transit areas. This could be the reason for better polyp detection than standard colonoscopy, as the latter depends on the operator's experience. In addition to the higher frame rate in second-generation capsule endoscopes, the larger angle of view of the two lenses and sufficient level of bowel preparation can also contribute to better results [10]. While the potential cost-effectiveness of CCE in the United States healthcare system is to be assessed, the choice of a noninvasive approach could lead to greater acceptability among the general population. This could be secondary to failure to expose proper polyp dimension, poor forceps access, or orientation. On the other hand, CCE holds the capability to record polyps from different aspects. This can also be modified due to luminal distention and magnification, as capsule photos are through the water while colonoscopy images are through the gas. Furthermore, the analytical software measuring polyp size has a 40% +- error range [9]. We propose that artificial intelligence (AI) could be employed to save the cost of data analysis and quicker results, including real-time options [13]. Real-time diagnostics can facilitate reduced expenses. CCE could be implemented in the United States CRC screening guidelines with further advancement on smaller, more efficient batteries, real-time computer-based analysis, and reporting.

## Conclusions

Our meta-analysis revealed higher per-patient sensitivity and specificity for detecting polyps ≥ 6 mm, and ≥ 10 mm; Further comparative data is needed. CCE holds screening potential secondary to the reduced complication rates. Technical improvements, like battery capacity, are required. The cost factor in undergoing capsule colonoscopy examinations is crucial, as in case of any suspicious polyps; patients would have to undergo subsequent colonoscopies. Our study does not address polyps < 6 mm. These polyps are mostly considered diminutive polyps, and an approach to managing diminutive polyps found on CCE is yet to be addressed. We conclude that addressing issues like incomplete capsule transit and bowel preparation quality is necessary.
